# The complete chloroplast genome of *Amana baohuaensis* (Liliaceae)

**DOI:** 10.1080/23802359.2020.1831989

**Published:** 2020-11-03

**Authors:** Long Wang, Xu Lu, Bangxing Han, Jingxin Chen, Jian Jin, Qinlin Lv, Hao Liu

**Affiliations:** a School of Traditional Chinese Medicine, China Pharmaceutical University, Nanjing, China; b Institute of Chinese Materia Medica, Hunan Academy of Chinese Medicine, Changsha, China; c College of Biological and Pharmaceutical Engineering, West Anhui University, Lu’an, China

**Keywords:** *Amana baohuaensis*, chloroplast genome, Liliaceae, phylogenetic analysis

## Abstract

*Amana baohuaensis* is a new species that was just named in 2019. Here, we obtained the complete chloroplast (cp) genome of *A. baohuaensis* using the Illumina paired-end sequencing technology. The cp genome has a typical quadripartite structure with 150,757 bp in length, containing a large single-copy (LSC) region of 81,757 bp, a small single-copy (SSC) region of 16,962 bp, and two inverted repeat (IR) regions of 26,019 bp. The total GC content is 36.73%, of which, the GC content of LSC, SSC and IR regions are 34.63%, 30.11% and 42.20%, respectively. The cp genome of *A. baohuaensis* contains 111 unique genes, including 78 protein-coding genes, 29 tRNA genes, and four rRNA genes. The Maximum Parsimony (MP) phylogenetic analysis suggested that *A. baohuaensis* had the closest relationship with *A. wanzhensis*, and all *Amana* species grouped together with high bootstrap support.

The genus of *Amana* is endemic to East Asia, which have six species in China (Tan et al. 2007; Han et al. 2014; Wang et al. 2019). This genus was previously placed in the genus *Tulipa*. For those genus plants were morphologically distinct from other tulips, they were then separated into a separate genus *Amana* (Wu et al. 2003; Tan et al. 2005; Li et al. 2017). The morphology of *Amana* genus plants is similar, and there are some difficulties in classification. With the development of gene sequencing technology, more and more researchers began to focus on the study of chloroplast (cp) genome. The cp genome is generally more conservative (Meng et al. 2018), which is of great significance for species identification and phylogeny (Xu et al. 2017; Gu et al. 2018). *Amana baohuaensis* B.X. Han, Long Wang & G.Y. Lu is a new species that was just named in 2019 and only occurs in East China, and the wild resource is few (Wang et al. 2019). In this study, the complete cp genome of *A. baohuaensis* was sequenced and analyzed, which provided a reference for systematic evolution and identification of this species.

The fresh leaves of *A. baohuaensis* were collected in Mt. Baohua, Jurong city, Jiangsu Province, China (32°07′31″ N, 119°05′24″ E). The voucher specimen was deposited in the Center of Herbarium, China Pharmaceutical University, Nanjing, China, under accession number WL194104. Whole genomic DNA was extracted from fresh leaves using Rapid Plant Genomic DNA Isolation Kit, Sangon Biotech (Shanghai) Co., Ltd. Then, the quality of DNA was checked using BioPhotometer Plus (Eppendorf, Germany). The good quality of DNA was sent to GENEWIZ (Suzhou, China) for sequencing. DNA sequencing was conducted on an Illumina Xten platform. Raw sequencing data were deposited in NCBI Sequence Read Archive (SRA) with the accession number PRJNA661717. Clean reads of the matched reference genes were extracted using the cp genome sequence of *A. edulis* (GenBank No. NC_034707.1) as a reference. These clean reads were assembled using NOVOPlasty v2.7.2 (Dierckxsens et al. 2016), and the assembled genome was annotated and analyzed using the online tool GeSeq (https://chlorobox.mpimp-golm.mpg.de/geseq.html), and manually adjusted. Finally, the cp genome sequence was deposited in GenBank with the accession number MT898423.

The complete cp genome of *A. baohuaensis* is 150,757 bp in length, which had the cyclic tetrad structure, containing a large single-copy (LSC) region of 81,757 bp, a small single-copy (SSC) region of 16,962 bp, and two inverted repeat (IR) regions of 26,019 bp. The total GC content is 36.73%, of which, the GC content of LSC, SSC and IR regions are 34.63%, 30.11% and 42.20%, respectively. A total of 111 genes were annotated in the cp genome of *A. baohuaensis*, including 78 protein-coding genes, 29 tRNA genes, and four rRNA genes.

For phylogenetic analysis, the complete cp genomes sequences of 18 species from Liliaceae were used to construct the phylogenetic tree, and *Allium obliquum* and *Veratrum japonicum* as outgroups. The sequences were aligned by MAFFT (Katoh and Standley 2013), and the sequences alignment result was examined and manually adjusted by BioEdit v. 7.0.9.0 (Hall 1999). Maximum Parsimony (MP) analysis was implemented using PAUP* v. 4.0 beta 10 (Swofford 2002) with 1000 bootstrap replicates to assess the reliability of the phylogenetic tree. The results showed that *A. baohuaensis* had the closest relationship with *A. wanzhensis*, and all *Amana* species grouped together with high bootstrap support (Figure 1). This study could provide valuable insight into systematic evolution, conservation and identification for species of *Amana*.

**Figure 1.**
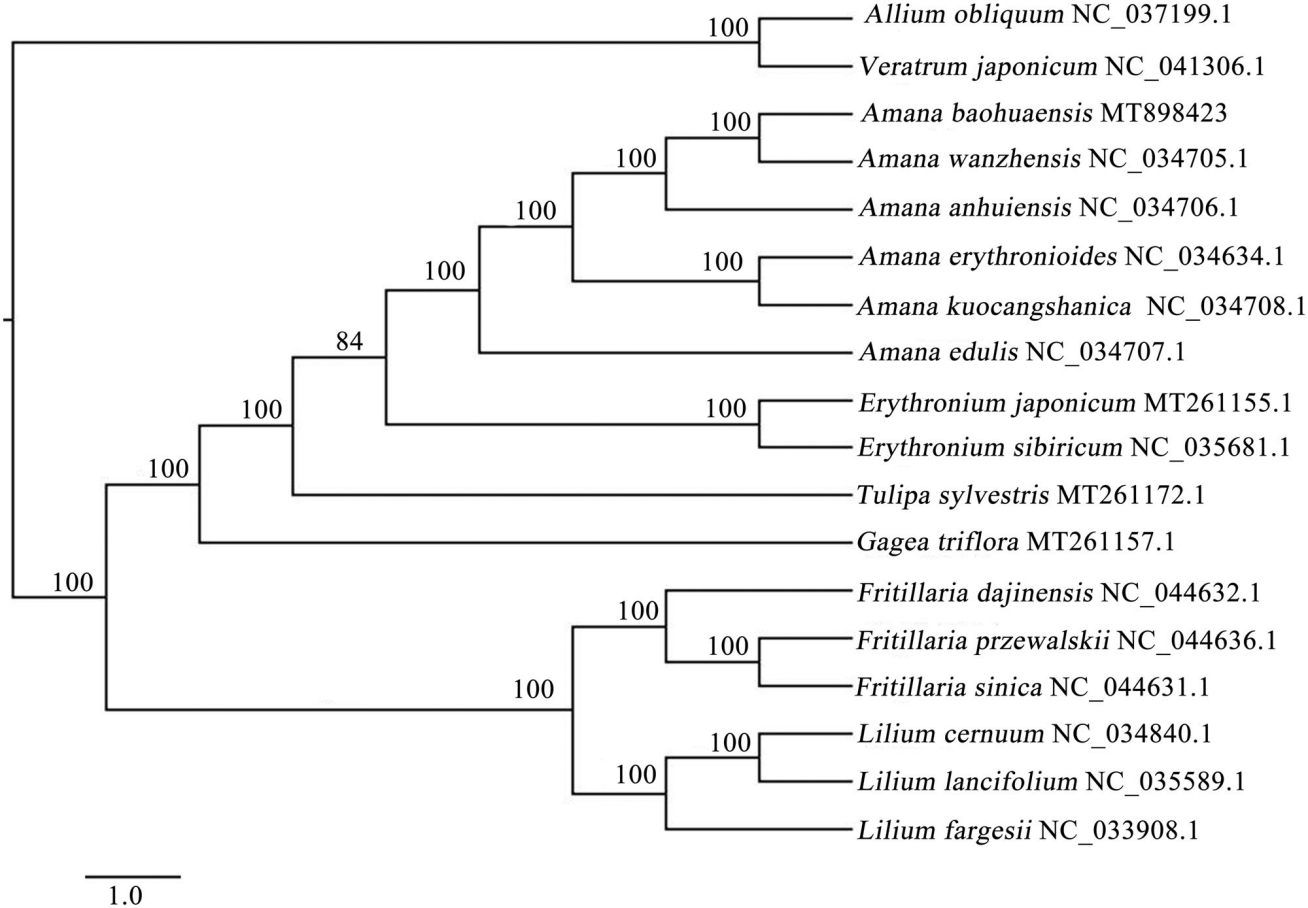
The Maximum Parsimony (MP) phylogenetic tree based on complete chloroplast genomes of 18 species. Numbers above the branches represent MP bootstrap (BS) values.

## Data Availability

The data that support the findings of this study are openly available in GenBank of NCBI at https://www.ncbi.nlm.nih.gov, reference number MT898423.
